# A 30-Year-Old Man With Primary Cardiac Angiosarcoma

**DOI:** 10.1016/j.jaccas.2021.03.009

**Published:** 2021-05-12

**Authors:** Nicholas Jex, Jonathan Farley, Sharmaine Thirunavukarasu, Amrit Chowdhary, Anshuman Sengupta, John Greenwood, Dominik Schlosshan, Sven Plein, Eylem Levelt

**Affiliations:** a University of Leeds, Multidisciplinary Cardiovascular Research Centre and Biomedical Imaging Science Department, Leeds Institute of Cardiovascular and Metabolic Medicine, Leeds, United Kingdom; b Department of Cardiology, Leeds Teaching Hospitals NHS Trust, Great George Street, Leeds, United Kingdom

**Keywords:** cardiac angiosarcoma, cardiac magnetic resonance, echocardiography, malignancy, imaging, positron emission tomography, CMR, cardiovascular magnetic resonance imaging, RA, right atrial, RCA, right coronary artery, RV, right ventricle

## Abstract

A previously fit and well 30-year-old man presented with palpitations, fever, and pleuritic chest pain. Multimodality imaging and histopathology confirmed the diagnosis of primary cardiac angiosarcoma. We present the details of the presentation, diagnostic process using multimodality imaging, and clinical management. (**Level of Difficulty: Beginner.)**

## Central Illustration

**Figure:**
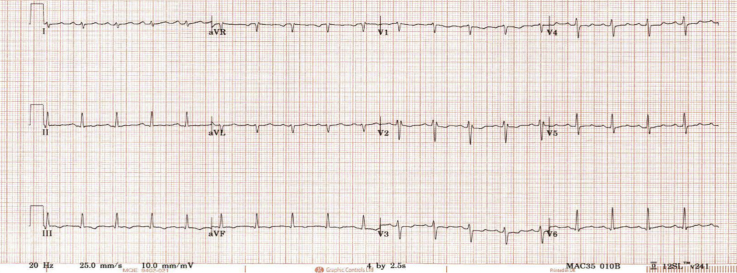

## History of Presentation

A previously fit and well 30-year-old man presented to the emergency department with palpitations, fever, and pleuritic chest pain. He also described an acute reduction in his exercise tolerance.

**Learning Objectives**

• To understand the 2 distinct clinical presentations of cardiac angiosarcoma and how these relate to the underlying morphological features.
• To review the diagnostic features of angiosarcoma on cardiovascular magnetic resonance imaging.
• To revisit therapeutic options and the importance of multidisciplinary care in this rare condition.

On examination, a resting tachycardia with heart rate of 110 beats/min and low-grade pyrexia of 37.8°C were detected. Oxygen saturations were 97% on room air, and blood pressure was normal. There were no signs of congestive cardiac failure or audible murmurs on pre-cordial auscultation.

## Medical History

The medical history was unremarkable.

## Differential Diagnosis

Given the history of pleuritic chest pain and fever and the timing of this presentation during the COVID-19 pandemic, coronavirus infection was the primary differential diagnosis. Venous pulmonary embolism and bacterial or other viral causes of pneumonia were also considered. Blood tests including D-dimer, electrocardiogram, and plain film chest x-ray film were ordered.

## Investigations

Full blood count showed a normocytic anemia. Although C-reactive protein (158 mg/l) and D-dimer (840 ng/ml) levels were raised, troponin I level (27.3 ng/l) was not raised. Liver function test results were normal.

COVID-19 swabs were negative.

**Figure 1:**
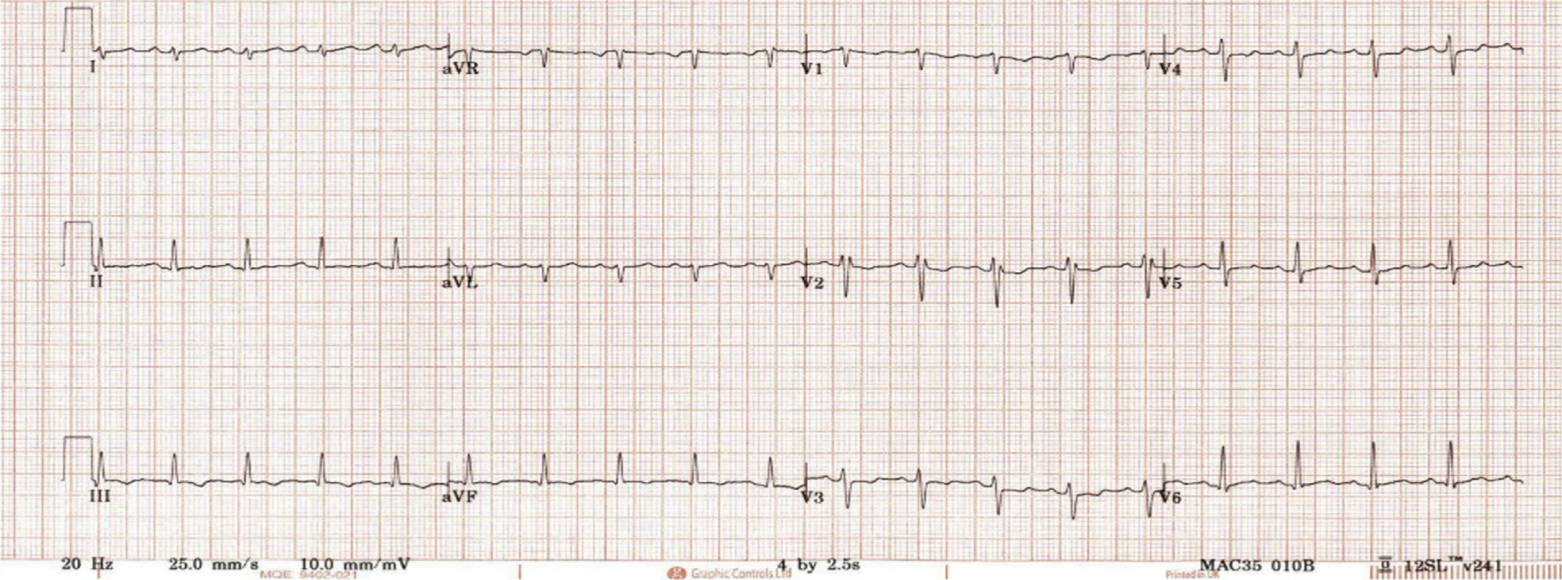
Electrocardiogram Showing Sinus Tachycardia

Electrocardiogram showed sinus tachycardia at 114 beats/min (Figure 1). Chest x-ray film findings were unremarkable.

**Figure 2:**
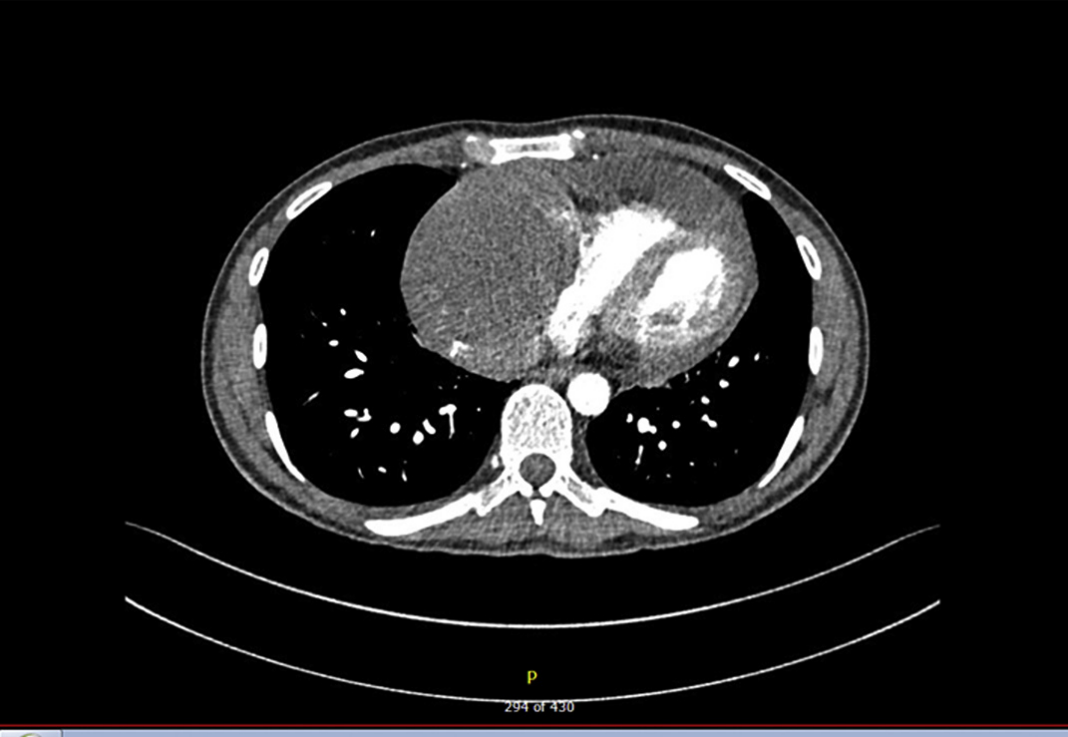
Computed Tomography Pulmonary Angiogram Showing an Anterior Mediastinal Mass

Given the presentation of pleuritic chest pain with sinus tachycardia and elevated D-dimer, computed tomography pulmonary angiogram was ordered. This excluded pulmonary embolism but demonstrated a large soft tissue mass in the anterior mediastinum (Figure 2).

Transthoracic echocardiogram confirmed the presence of a large hypoechoic right atrial (RA) mass. The patient was admitted to the ward for further assessment.

**Figure 3:**
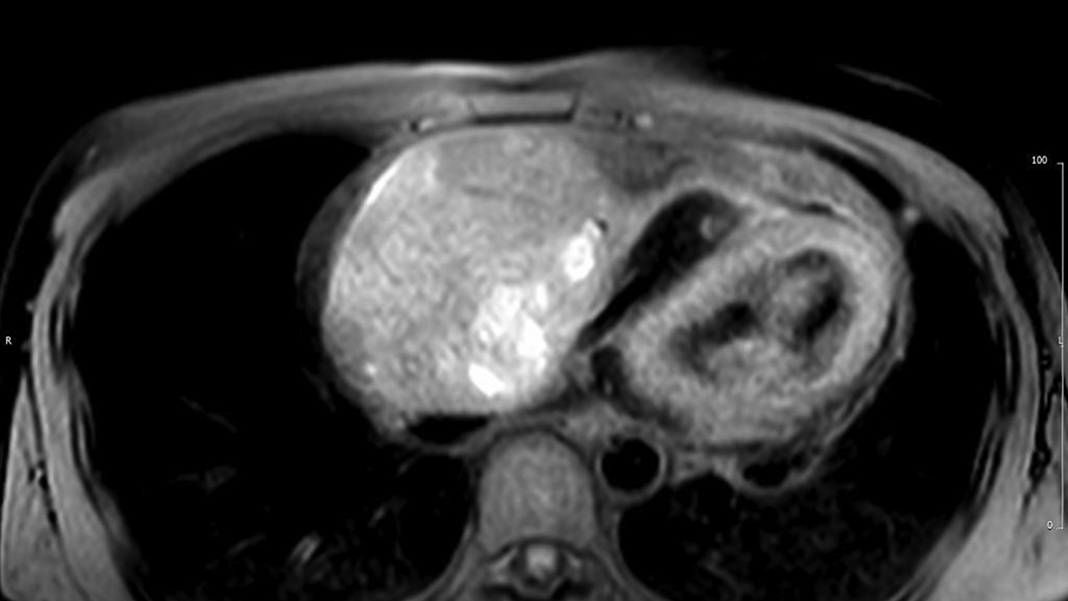
T_1_-Weighted Cardiac Magnetic Resonance Image Showing the Right Atrial Mass

**Figure 4:**
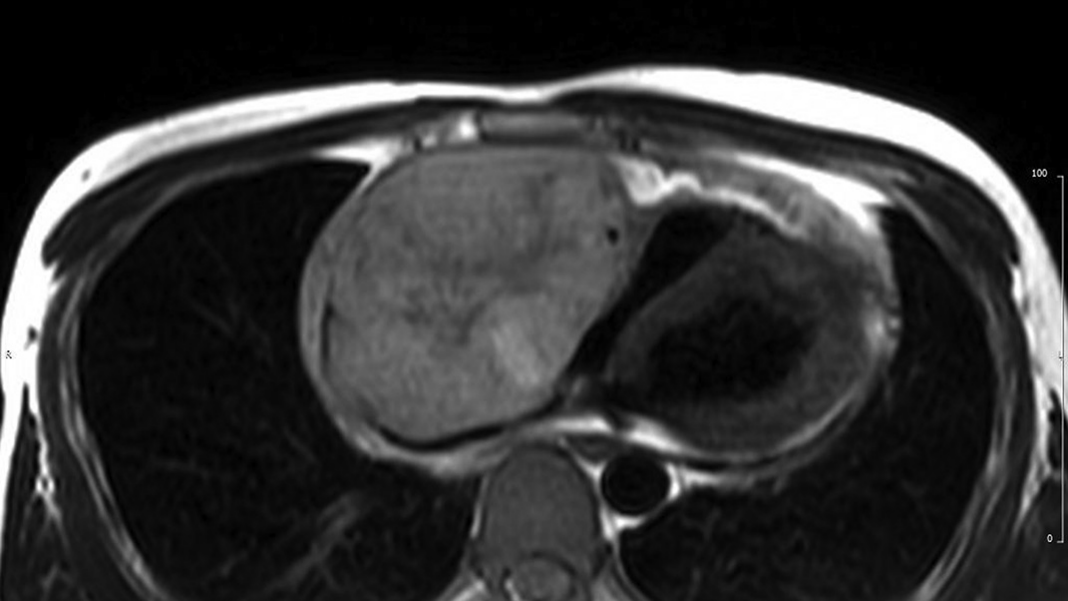
T_2_-Weighted Cardiac Magnetic Resonance Image Showing the Right Atrial Mass

**Figure 5:**
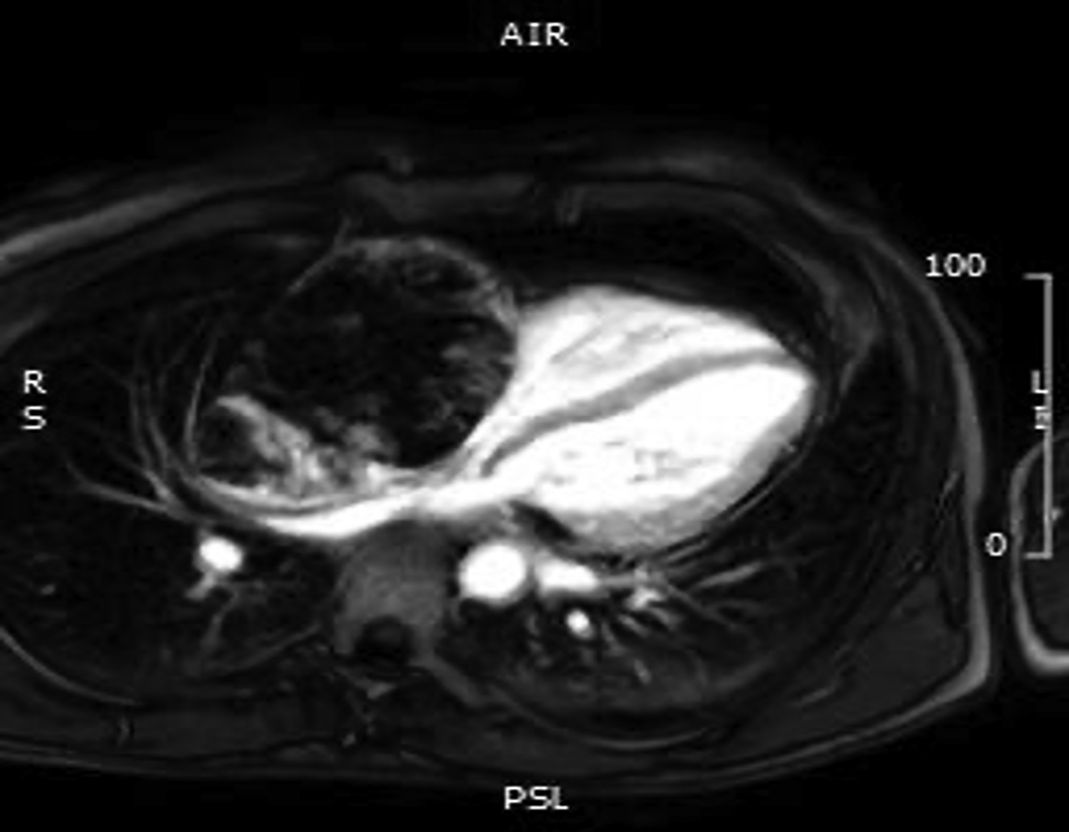
Early Gadolinium Enhanced Cardiac Magnetic Resonance Image of the Right Atrial Mass

**Figure 6:**
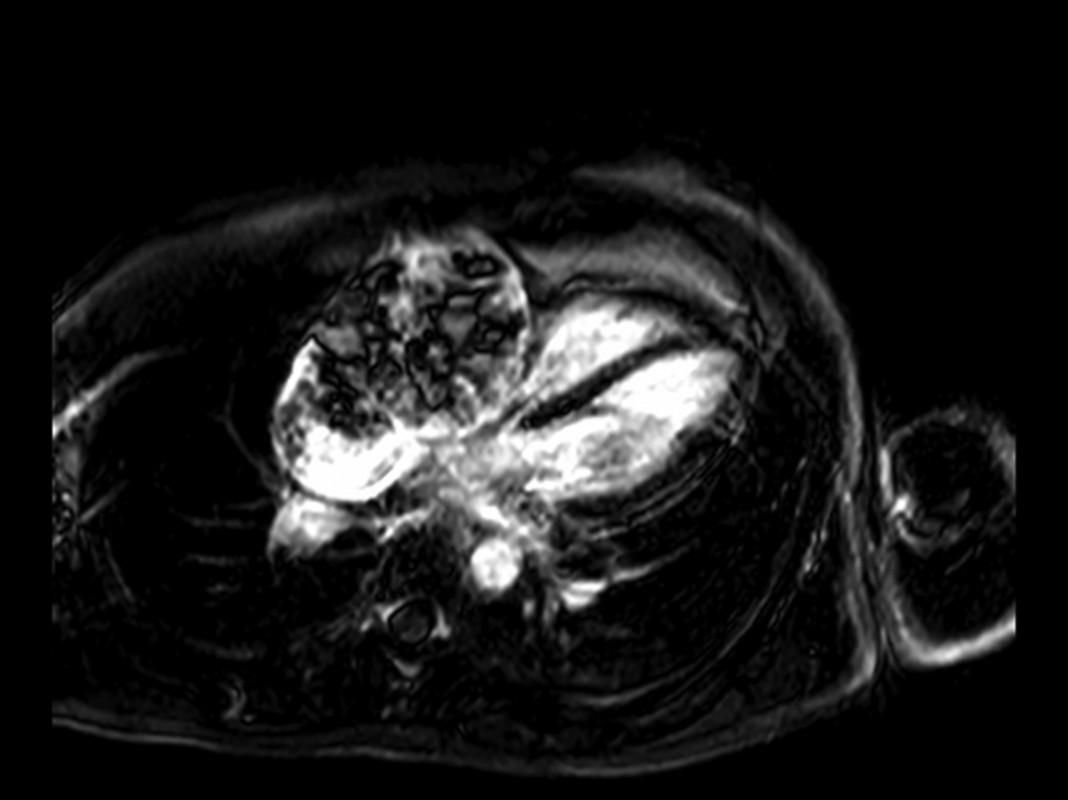
Late Gadolinium Enhanced Cardiac Magnetic Resonance Image of the Right Atrial Mass

Cardiac magnetic resonance (CMR) confirmed the presence of an RA mass measuring 12 × 8 cm, filling >90% of the chamber lumen (Video 1). The mass was invading the RA lateral wall, atrioventricular groove, and anterior tricuspid valve cusp and compressing the inferior vena cava. Global pericardial effusion with a maximal diameter measuring 2 cm around the right ventricle (RV) free wall was detected. The mass showed heterogeneous signal intensity on T_1_- and T_2_-weighted imaging (Figures 3 and 4) and on early and late gadolinium enhancement imaging (Figures 5 and 6). First-pass perfusion imaging with a standard rest perfusion sequence demonstrated regions of heterogeneous enhancement with visually higher perfusion at the margins than at the core of the lesion, likely due to regional variations in vascularity and regions of necrosis at the core of the mass (Video 2). Left ventricle systolic function was globally mildly impaired, with an ejection fraction of 50%. The base of the RV free wall was invaded by the mass; mid and apical RV function was preserved. A preliminary diagnosis of cardiac angiosarcoma was suggested based on CMR appearances.

**Figure 7:**
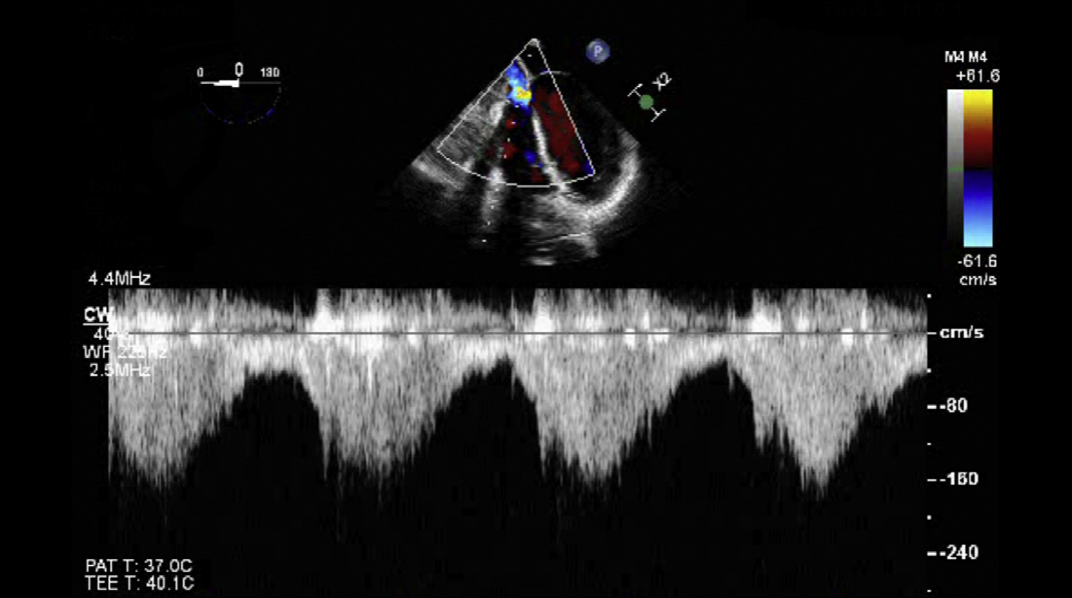
Transesophageal Echocardiography Showing no Functional Tricuspid Valve Stenosis

No functional tricuspid valve stenosis was detected on transesophageal echocardiography (Figure 7).

**Figure 8:**
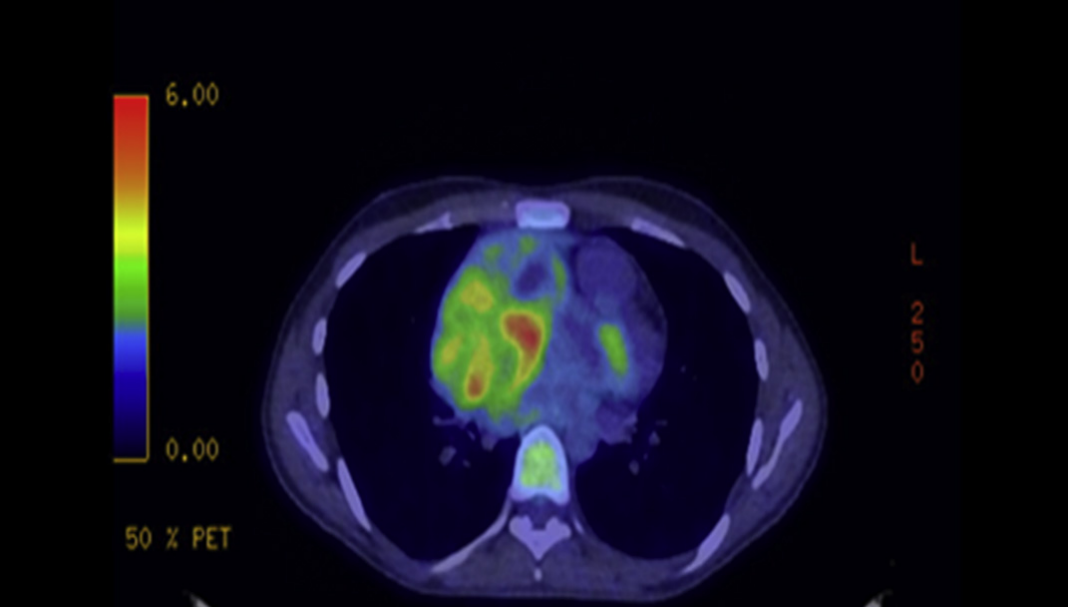
Fluorodeoxyglucose–Positron Emission Tomography Showing Moderate Uptake in the Right Atrial Mass

**Figure 9:**
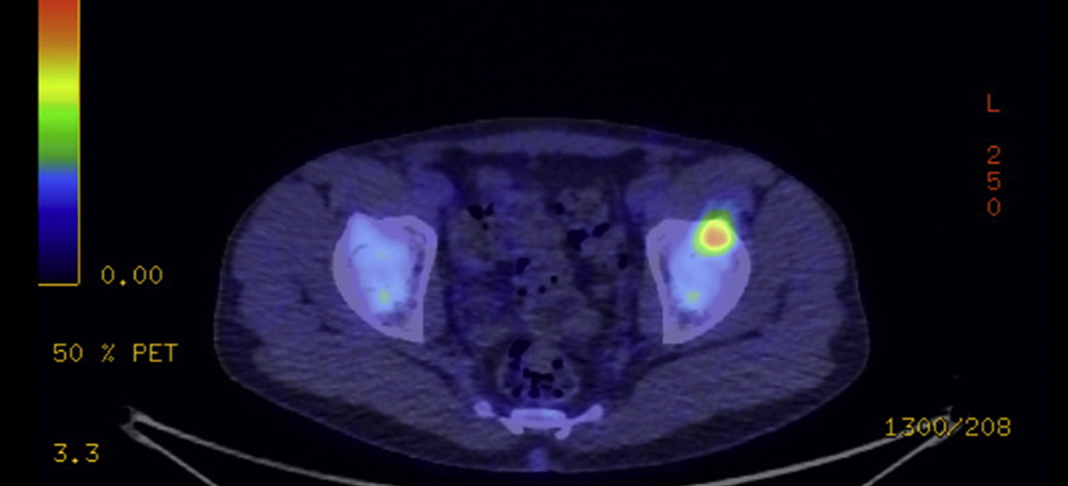
Fluorodeoxyglucose–Positron Emission Tomography Showing Uptake in the Anterior Iliac Spine Compatible With Bony Metastases

The RA mass demonstrated moderate uptake on fluorodeoxyglucose–positron emission tomography scan. Lymph node involvement was seen with low-grade hyperenhancement of the right internal mammary nodes. There was focal fluorodeoxyglucose uptake in the right iliac crest and left anterior iliac spine compatible with bony metastases (Figures 8 and 9).

Transjugular core biopsy was performed for histopathologic analysis. This showed a spindle cell tumor, staining strongly with CD34 and ERG (erythroblast transformation-specific related gene), confirming the radiological suspicion of angiosarcoma.

Coronary angiography was performed in preparation for debulking surgery. This showed a highly vascular tumor with supply from the right coronary artery (RCA) and proximal circumflex artery. There was also a mass effect compressing the RCA (Videos 3 and 4).

## Management

Because of the presence of widespread metastatic disease and the extent of regional spread and size of the tumor, curative treatment was not considered to be possible by the multidisciplinary team.

The patient underwent successful debulking surgery with reconstruction of the RA wall and grafting of the RCA. Post-operative transesophageal echocardiography showed preserved left ventricle systolic function with mildly dilated and impaired RV systolic function (Videos 5, 6, 7, 8, and 9).

Following surgery, the patient underwent palliative chemotherapy with doxorubicin and palliative radiotherapy at the sites of bony metastases.

## Discussion

Angiosarcoma is the most common primary cardiac malignancy in adulthood, comprising 9% of all primary cardiac tumors (1). Angiosarcomas typically occur in the RA (90%) and more commonly affect men (2). Presentation is typically between the third and fifth decades of life (3) and carries a poor prognosis because of the rapid growth of the tumor and frequent presence of metastases at the time of diagnosis (2). Histologically, angiosarcomas show rapidly proliferating infiltrating anaplastic cells derived from the vasculature. Tumors have well-differentiated vascular channels combined with poorly differentiated areas of epithelioid and spindle cells (2).

There are typically 2 distinct clinical presentations, reflecting the 2 common morphological variants of the tumor (1). The focal well-defined form, as seen in our case, with a mass protruding into the RA presents with symptoms related to severe intracavity obstruction. These patients most commonly present with dyspnea and chest pain. The diffuse form of the disease rapidly invades the RV and the pericardium, resulting in heart failure or cardiac tamponade due to the tumor’s permeating and destructive nature (1).

The CMR features of angiosarcoma are characterized by a heterogenous RA mass with or without pericardial involvement. Heterogeneity on T_1_- and T_2_- weighted images and LGE reflects areas of tumor tissue, necrosis, and hemorrhage (1). Arterial phase enhancement on first-pass perfusion imaging reflects the vascularity of the tumor. Our case reveals that large tumors may have heterogenous perfusion with higher vascularity only at the margins, likely due to the presence of necrosis at the core of the lesion.

Although the presenting symptoms of breathlessness and palpitations are reported in similar frequencies for both benign and malignant RA masses, the size of the mass (>5 cm), invasion of the RV base, variable tissue intensity on T_1_- and T_2_-weighted CMR images, high perfusion uptake, and accompanying moderate pericardial effusion indicated a malignant tumor. Accordingly, metastatic malignancies and lymphomas were considered as the most likely differential diagnosis. Lymphomas, the primary differential, are typically homogenous and isointense on T_1_- and T_2_-weighted images with minimal contrast uptake on LGE (1).

The prognosis of angiosarcoma is poor, with a median survival of 14 months, reducing to 6 months in metastatic disease (3). Palliative treatment with surgical debulking and chemoradiotherapy may offer some prognostic benefit (4). Anthracyclines, ifosfamide, and taxanes are the most commonly used agents (5). Immunotherapy with recombinant interleukin 2 has been used with some prognostic benefit (6).

In our case, the multidisciplinary team decision was for surgical debulking to provide the best option for palliative care, given the degree of RA cavity obstruction, and to allow time for chemotherapy administration.

Because of the rare incidence of cardiac angiosarcomas, there is a dearth of evidenced-based guidelines, and currently, no standardized treatment algorithm for cardiac angiosarcoma exists (3).

## Follow-Up

Following surgery and palliative chemoradiotherapy, repeat imaging showed significant metastatic disease progression within the lungs and pelvis. In addition to doxorubicin and single-slice radiotherapy, hospice services were engaged. The patient died 4 months following the initial diagnosis.

## Conclusions

This case demonstrates the typical presentation of primary cardiac angiosarcoma and its associated features on multimodality imaging. Multidisciplinary care of this relatively rare tumor is important, which reflects the lack of consensus guidelines on management.

## Funding Support and Author Disclosures

The authors have reported that they have no relationships relevant to the contents of this paper to disclose.

## Appendix

Supplemental Video 1 Cardiovascular magnetic resonance imaging, four chamber horizontal long axis view cine, showing right atrial mass and associated pericardial effusion

Supplemental Video 2 Cardiovascular magnetic resonance imaging, first pass perfusion, showing regional variations in tumour vascularity

Supplemental Video 3 Coronary angiography, anterior oblique view, showing vascular supply to the tumour from right coronary artery (RCA) with associated mid vessel compression of the RCA from the tumour

Supplemental Video 4 Coronary angiography, right anterior oblique caudal view, showing vascular supply to the tumour from the proximal circumflex artery

Supplemental Video 5 Pre-operative trans oesophageal echocardiography, four chamber view, showing restricted flow within the right atrium

Supplemental Video 6 Pre-operative trans oesophageal echocardiography, transgastric view, showing near total right atrial cavity obstruction from the angiosarcoma

Supplemental Video 7 Trans oesophageal echocardiography, 3D reconstructed image, showing right atrial angiosarcoma

Supplemental Video 8 Post-operative trans oesophageal echocardiography, trans-gastric view, showing reconstructed right atrium following tumour excision

Supplemental Video 9 Post-operative trans oesophageal echocardiography, trans-gastric view
